# Optimal Serum Ferritin Levels for Iron Deficiency Anemia during Oral Iron Therapy (OIT) in Japanese Hemodialysis Patients with Minor Inflammation and Benefit of Intravenous Iron Therapy for OIT-Nonresponders

**DOI:** 10.3390/nu10040428

**Published:** 2018-03-29

**Authors:** Kazuya Takasawa, Chikako Takaeda, Takashi Wada, Norishi Ueda

**Affiliations:** 1 Department of Internal Medicine, Division of Nephrology, Public Central Hospital of Matto Ishikawa, Ishikawa 9248588, Japan; takaeda@imcc-med.com; 2 Department of Nephrology, Kanazawa University; Kanazawa, Ishikawa 9208641, Japan; twada@m-kanazawa.jp; 3 Department of Pediatrics, Public Central Hospital of Matto Ishikawa, Ishikawa 9248588, Japan

**Keywords:** ferritin, hemodialysis, hepcidin-25, inflammation, iron deficiency anemia, oral iron therapy

## Abstract

**Background:** We determined optimal serum ferritin for oral iron therapy (OIT) in hemodialysis (HD) patients with iron deficiency anemia (IDA)/minor inflammation, and benefit of intravenous iron therapy (IIT) for OIT-nonresponders. **Methods:** Inclusion criteria were IDA (Hb <120 g/L, serum ferritin <227.4 pmol/L). Exclusion criteria were inflammation (C-reactive protein (CRP) ≥ 5 mg/L), bleeding, or cancer. IIT was withheld >3 months before the study. ΔHb ≥ 20 g/L above baseline or maintaining target Hb (tHB; 120–130 g/L) was considered responsive. Fifty-one patients received OIT (ferrous fumarate, 50 mg/day) for 3 months; this continued in OIT-responders but was switched to IIT (saccharated ferric oxide, 40 mg/week) in OIT-nonresponders for 4 months. All received continuous erythropoietin receptor activator (CERA). Hb, ferritin, hepcidin-25, and CERA dose were measured. **Results:** Demographics before OIT were similar between OIT-responders and OIT-nonresponders except low Hb and high triglycerides in OIT-nonresponders. Thirty-nine were OIT-responders with reduced CERA dose. Hb rose with a peak at 5 months. Ferritin and hepcidin-25 continuously increased. Hb positively correlated with ferritin in OIT-responders (*r* = 0.913, *p* = 0.03) till 5 months after OIT. The correlation equation estimated optimal ferritin of 30–40 ng/mL using tHb (120–130 g/L). Seven OIT-nonresponders were IIT-responders. **Conclusions:** Optimal serum ferritin for OIT is 67.4–89.9 pmol/L in HD patients with IDA/minor inflammation. IIT may be a second line of treatment for OIT-nonreponders.

## 1. Introduction

Iron deficiency anemia (IDA) is a common problem, which causes resistance to erythropoietin-stimulating agents (ESAs), is associated with patients on chronic hemodialysis (HD), and increases morbidity and mortality, whereas correction of anemia improves these events in HD patients [1]. IDA is generally defined by serum ferritin of <67.4 pmol/L and transferrin saturation (TSAT) <16%, while higher cutoffs of serum ferritin and TSAT are used to define IDA under inflammatory conditions such as chronic kidney disease (CKD) [2]. The Kidney Disease Improving Global Outcome (KDIGO) guidelines recommend that iron therapy should be initiated if CKD patients have serum ferritin ≤1123.5 pmol/L and TSAT ≤30% [3]. In Europe, it is recommended that serum levels of ferritin should be maintained at 898.8–1348.2 pmol/L for the management of IDA in HD patients [4]. However, the Japanese Society for Dialysis Therapy (JSDT) guidelines use more conservative criteria for IDA (serum ferritin <227.4 pmol/L and TSAT <20%) probably due to lower prevalence of inflammation in the Japanese HD patients [5]. In fact, prevalence of increased C-reactive protein (CRP) levels, a marker of inflammation, in HD patients was higher in Western countries than in Japan [6,7] and of catheter use for HD and obesity, which can increase inflammation, was lower in HD patients of Japan than those of Western countries [6].

Intravenous iron therapy (IIT) has been proposed to have superior benefit over oral iron therapy (OIT) for the management of IDA and efficient maintenance of target hemoglobin (tHb) in HD patients [8]. Recently, the majority of HD patients receiving IIT and ESAs have been shown to have hepatic iron overload evaluated by magnetic resonance imaging (MRI) [9,10]. A risk of hospitalization, cardiovascular events, infection, and mortality was significantly higher in HD patients receiving higher doses of IIT [11,12] and ESAs [13] than in those receiving lower doses [14]. Mortality was significantly higher in HD patients receiving an IV iron dose of >300 mg/month than in those receiving iron dose of <299 mg/month [11]. The MRI study suggested that the standard maximal amount of iron infused per month should be lowered to <250 mg/month in order to reduce a risk of iron overload and allow safer use of parenteral iron products [9]. These findings may call for a revision of clinical guidelines of the management of IDA in patients with chronic kidney disease (CKD), especially in HD patients, including the root and dose of iron supplementation.

Iron supplementation with avoidance of iron overload is crucial for the management of IDA in HD patients. For the purpose of appropriate management of IDA in CKD patients, rapid, accurate and noninvasive methods for monitoring iron stores in the body are mandatory, but unfortunately not available except the measurement of total body iron by MRI. Serum ferritin is a most commonly used and reliable biomarker of iron status in the absence of inflammation [2]. Serum levels of ferritin were positively correlated with liver iron content in HD patients [9,10]. High serum levels of ferritin reflected iron overload [10], resulting in iron toxicity and high mortality in CKD patients [15]. However, as acute reactants, serum ferritin and CRP are up-regulated by inflammation, which is frequently associated with CKD [2,16]. Thus, interpretation of data for serum ferritin should be with caution. Furthermore, both parameters were frequently increased in HD patients with functional IDA (FIDA) [17], accompanied by high inflammation, and HD patients with FIDA required higher dose of IIT than those without [18]. On the contrary, lower levels of CRP were predictive of a greater response to OIT in HD patients [19]. Taken together, these data suggest that therapeutic strategy for IDA should differ between HD patients with and without high inflammation.

Serum levels of ferritin were higher in HD patients treated with IIT than those with OIT [20], suggesting a possible risk of iron overload in HD patients receiving IIT compared to those receiving OIT. OIT is less toxic than IIT [8,21] and may reduce a risk of iron overload which leads to cardiovascular events, infection and mortality [22]. OIT has recently been shown to be as effective as IIT for the management of IDA in non-dialysis CKD [21] and HD patients [23,24,25,26] with relatively lower serum levels of ferritin and normal CRP. In the former study [21], IIT was associated with an increased risk of serious adverse events, including cardiovascular events and infectious disease. We have previously reported that OIT was beneficial for IDA in HD patients with minor inflammation and that ferritin and hepcidin-25 could be predictive of the OIT response [25]. However, it remains elusive what levels of serum ferritin are optimal for the management of IDA during iron supplementation in CKD patients to reduce a risk of iron overload-related adverse effects [2]. This prospective study was thus undertaken to determine the following, (1) what levels of serum ferritin are optimal for OIT in HD patients with IDA and minor inflammation receiving a continuous erythropoietin receptor activator (CERA)? And (2) whether IIT could be a second line of treatment for IDA in HD patients who are refractory to OIT.

## 2. Materials and Methods

Inclusion criteria of the study were adult HD patients (≥18 years of age) and IDA (hemoglobin; Hb < 120 g/L and ferritin <227.4 pmol/L). Exclusion criteria were inflammation as defined by the presence of C-reactive protein (CRP) ≥ 5 mg/L, bleeding, cancer or poor adherence. Iron supplementation was withheld >3 months prior to the study.

**Figure 1:**
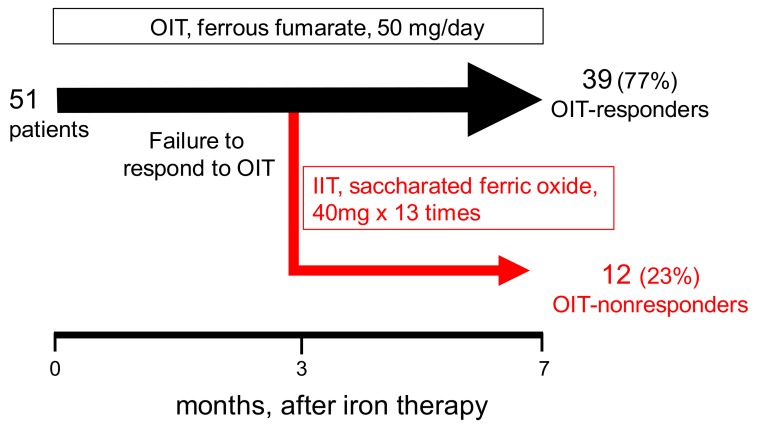
Protocol of iron therapy. Fifty-one consecutive hemodialysis (HD) patients with iron deficiency anemia (IDA) and minor inflammation were first treated with oral ferrous fumarate (50 mg/day). At 3 months after oral iron therapy (OIT), the patients were classified into two groups; OIT-responders and OIT-nonresponders. OIT was continued in 39 OIT-responders for another 4 months. OIT was switched to intravenous iron therapy (IIT; saccharated ferric oxide: 40 mg × 13 times for another 4 months) in the remaining 12 OIT-nonresponders. All patients simultaneously received a continuous erythropoietin receptor activator (CERA) during the study period.

At the initiation of the present study, there were 70 patients on maintenance HD in our hospital. Of these, 51 consecutive HD patients with IDA fulfilled the inclusion criteria and were enrolled in the study. This study was non-randomized and prospective study performed at our single center. All patients received oral ferrous fumarate (50 mg/day) for the first 3 months (Figure 1). The response to OIT was determined at 3 months after OIT since serum ferritin started to rise within this period (see in Section 3). At this time point, the patients were classified into two groups; OIT-responders and OIT-nonresponders. In OIT-responders, oral ferrous fumarate was continued for another 4 months. In OIT-nonresponders, OIT was switched to IIT (40 mg of saccharated ferric oxide), which was given 13 times during another 4 months. The dose and duration of this IIT protocol has been recommended by the JSDT guidelines [5]. All patients simultaneously received a CERA (epoetin β pegol) during the study period. The response to OIT or IIT was defined by the change in Hb levels before and after iron supplementation (ΔHb) of ≥200 g/L above baseline or maintenance of target Hb (tHb; 120~130 g/L). Since ΔHb of ≥10 g/L was used as an index of the response to iron therapy [4], the number of the patients with ΔHb of <20 g/L, but ≥10 g/L were also presented.

The levels of Hb and serum levels of ferritin, as measured by standard laboratory methods, were analyzed every month after the initiation of iron supplementation. Serum levels of hepcidin-25, a key regulatory hormone of iron metabolism, were measured by liquid chromatography tandem-mass spectrometry (LC-MS/MS) at 0, 6, and 7 months after iron supplementation as described in our previous study [25]. To determine optimal levels of serum ferritin in OIT-responders, correlation between the levels of Hb and serum ferritin was determined, and then optimal serum levels of ferritin were calculated by the correlation efficient using tHb of 120–130 g/L. The dose of CERA was measured at 0, 3, and 6 months after iron supplementation, and compared between groups. Written informed consent was obtained from all participants prior to entering the study. Our institutional research and ethics review board approved the study (approval code: 28-1).

### Statistical Analysis

Data are expressed as median (interquartile) in table, mean ± standard deviation (SD) in text and mean ± standard error of the mean (SEM) in figures. Comparison of two nonparametric data groups was performed using the Mann–Whitney U test. Comparison of nonparametric data in 3 groups was determined by Tukey–Kramer test, and that of two proportions was performed using the Fisher’s exact test. The linear correlation between Hb and serum ferritin levels was determined using Pearson’s correlation coefficient test. A *p* value < 0.05 was considered significant.

## 3. Results

Of the 51 HD patients, 39 patients (77%) responded to OIT (OIT-responders), and the remaining 12 patients (OIT-nonresponders) failed to respond to a 3-month-course of OIT (Figure 1 and Table 1). Demographic and baseline laboratory data before starting OIT in both the OIT-responders and the OIT-nonresponders are summarized in Table 1. There was no difference between the two groups in the variables except low Hb levels (*p* < 0.05) and high levels of serum triglycerides (*p* < 0.05) in the OIT-nonresponders. In the absence of apparent inflammation, serum hepcidin-25 is positively correlated with Hb till iron-repletion state is achieved. Thus, as Hb, the baseline hepcidin-25 levels tended to be lower in the OIT-nonresponders than in the OIT-responders. When ΔHb ≥ 10 g/L was used as criteria for the response to iron therapy, prevalence of OIT response was 44 of 51 patients (86.3%). At the end of the study, only one IIT-nonresponder achieved ΔHb <20 g/L but ≥10 g/L.

**Figure 2:**
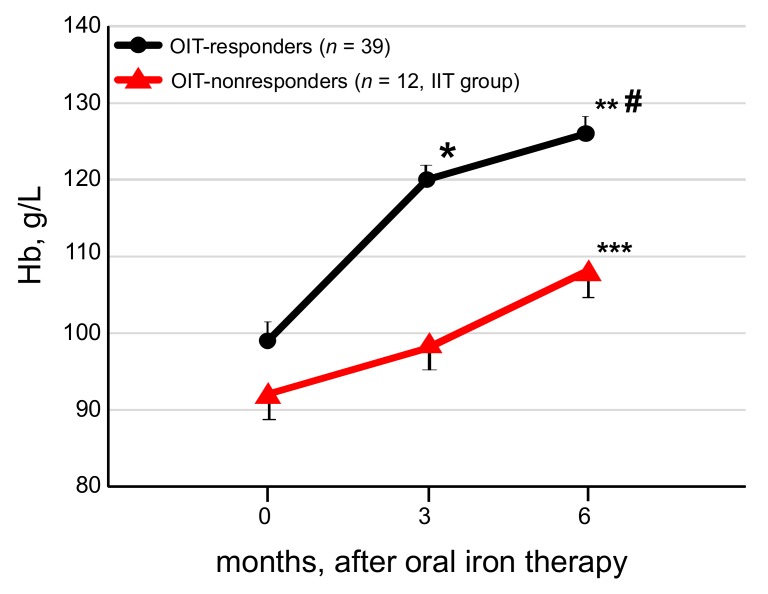
Change of Hb levels in OIT-responders and OIT-nonresponders. The Hb levels were increased at 3 and 6 months after OIT in OIT-responders. In OIT-nonresponders, the Hb levels remained unchanged at 3 months after OIT but increased with IIT at the end of the study as a whole IIT-group. Data are expressed as mean ± standard error of the mean (SEM). Comparison of two nonparametric data groups was analyzed by the Mann-Whitney U test. * *p* < 0.01, vs. data at 0 month, ** *p* < 0.01, vs. data at 3 months, *** *p* < 0.05, vs. data at 3 months, # *p* < 0.05, vs. OIT-nonresponders.

In the OIT-responders, mean levels of Hb rose from a baseline of 99 ± 11 g/L to 120 ± 6 g/L (*p* < 0.05) at 3 months and 126 ± 12 g/L (*p* < 0.05) at 6 months after OIT (Figure 2). The ΔHb at 3 and 6 months after OIT were 17 ± 6 g/L (*p* < 0.01) and 27 ± 19 g/L (*p* < 0.01), respectively, and the ΔHb at 6 months was higher (*p* < 0.01) than that at 3 months after OIT. The ΔHb were 30 ± 11 g/L at 3 months and 40 ± 13 g/L at 6 months after OIT in the 21 OIT-responders with ΔHb ≥ 20 g/L, and 10 ± 6 g/L at 3 months and 12 ± 11 g/L at 6 months after OIT in the remaining 18 OIT-responders who achieved the tHb but ΔHb < 20 g/L. In the 12 OIT-nonresponders, the baseline Hb was 92 ± 11 g/L, the Hb was 98 ± 8 g/L at 3 months after OIT, and the ΔHb remained unchanged (6 ± 12 g/L). In the OIT-nonresponders (*n* = 12), after switching to IIT, the ΔHb significantly rose at 6 months after OIT (16 ± 20 g/L, *p* < 0.01) compared to that at 3 months after OIT (6 ± 12 g/L). 

In the OIT-responders, the CERA dose significantly decreased from baseline of 135 ± 45 μg/4 weeks to 111 ± 49 μg/4 weeks (*p* < 0.05) at the end of the study, while it remained unchanged in the OIT-nonresponders (152 ± 57 μg/4 weeks at baseline vs. 160 ± 56 μg/4 weeks at 6 months). 

**Figure 3:**
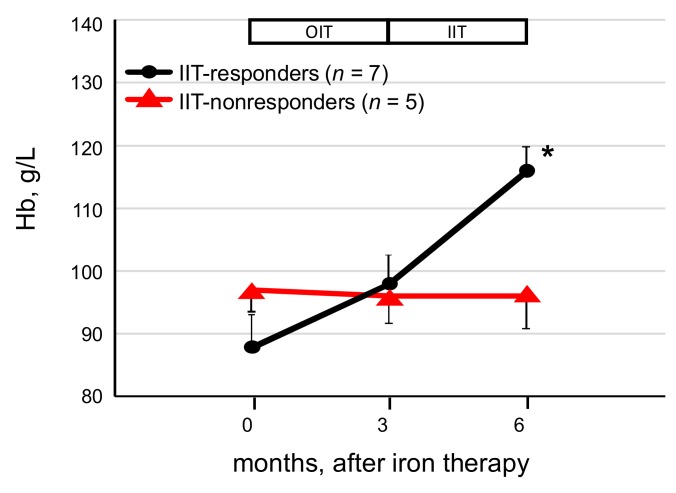
Change of Hb levels in IIT group before and after IIT. Of the 12 OIT-nonresponders, the levels of Hb were significantly increased in seven (58.3%) of the 12 patients at the end of the study, whereas the Hb levels remained unchanged in the five IIT-nonresponders. Data are expressed as mean ± SEM. Comparison of two nonparametric data groups was analyzed by the Mann–Whitney U test. * *p* < 0.01, vs. IIT-nonresponders.

Of the 12 OIT-nonresponders receiving IIT, seven patients (58%, IIT-responders) responded to IIT, and the remaining 5 failed to respond (IIT-nonresponders, Figure 3). In the IIT-responders, the ΔHb significantly rose from the value for 11 ± 13 g/L at 3 months to 17 ± 5 g/L (*p* < 0.05) at 6 months, respectively, whereas ΔHb at 3 and 6 months after iron therapy was 1 ± 9 g/L and −1 ± 10 g/L in the IIT-nonresponders.

**Figure 4:**
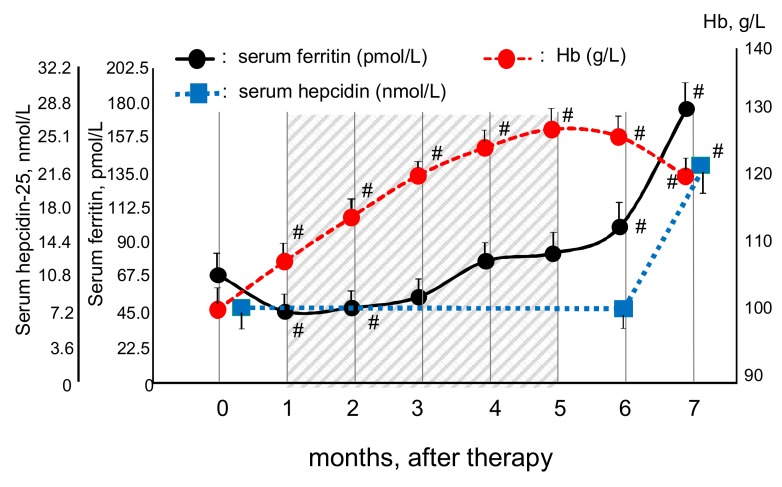
Sequential change in the levels of Hb, serum ferritin and hepcidin-25 in OIT-responders during OIT. The levels of Hb rose linearly with a peak at 5 months after OIT and then slightly decreased at the end of the study. Serum levels of ferritin were decreased from baseline at 1 month, and then rose continuously from 2 to 7 months after OIT. Serum levels of hepcidin-25 were similar to baseline at 6 months but significantly increased at the end of the study. Serum levels of hepcidin-25 were positively correlated with those of ferritin in the OIT-responders (*r* = 0.869, *p* = 0.0002). Data are expressed as mean ± SEM. Comparison of 2 means was determined by the Man–Whitney U test. # *p* < 0.01, vs. data at 0 month.

To determine optimal serum levels of ferritin during OIT for the management of IDA in HD patients, we first analyzed sequential changes in the levels of Hb, serum ferritin and hepcidin-25 during OIT in the OIT-responders (Figure 4). The levels of Hb rose linearly with a peak at 5 months, and then slightly decreased till the end of the study. Serum levels of ferritin were decreased from the baseline at 1 month, and then rose continuously from 2 to 7 months after OIT. Serum levels of hepcidin-25 at 6 months (8.0 ± 7.2 nmol/L) were similar to the baseline data (8.1 ± 9.1 nmol/L) but significantly increased (21.6 ± 16.2 nmol/L, *p* < 0.001) at the end of the study. Serum levels of hepcidin-25 were positively correlated with those of ferritin in the OIT-responders (*r* = 0.852, *p* < 0.0001) at the start of OIT. 

**Figure 5:**
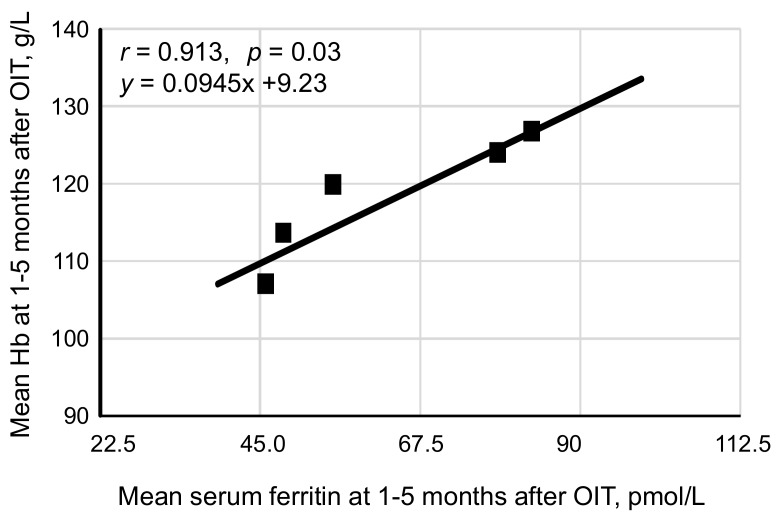
Correlation of Hb and serum ferritin between 1 and 5 months after OIT in OIT-responders. Ccorrelation between the levels of Hb and serum ferritin was analyzed in 39 OIT-responders using mean values for Hb and serum ferritin at every month obtained from OIT-responders as a whole. The levels of Hb were positively correlated with serum levels of ferritin till 5 months after OIT in 39 OIT-responders. The correlation equation calculated by Pearson’s correlation coefficient test was y = 0.0945x + 9.23, where y = Hb: x = serum ferritin.

We next examined correlation between the levels of Hb and serum ferritin in the OIT-responders using mean values of Hb and serum ferritin in the OIT responders at every month during the study period. Despite no correlation was found between these parameters during 4–7 months after OIT, the levels of Hb were positively correlated with those of serum ferritin during 1 to 5 months after OIT (*r* = 0.913, *p* = 0.03, Figure 5). The correlation equation calculated by Peason’s correlation coefficient test was y = 0.0945x + 9.23, where y = Hb and x = serum ferritin. Based on this equation, optimal levels of serum ferritin for the management of IDA were estimated to be 67.4–89.9 pmol/L when the tHb was 120–130 g/L.

**Figure 6:**
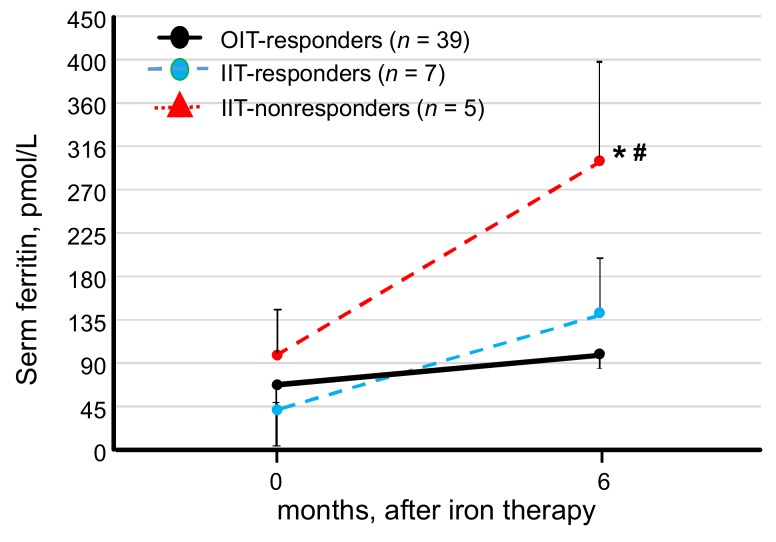
Change in serum levels of ferritin at 0 and 6 months after initiation of the study in OIT-responders, IIT-responders and IIT-nonresponders. Serum levels of ferritin were significantly higher in the IIT-nonresponders than in the OIT-responders and the IIT-responders, whereas there was no difference in serum ferritin levels between the OIT-responders and the IIT-responders. Comparison of three nonparametric data groups was analyzed by the Tukey–Kramer test. * *p* < 0.05, vs. IIT-responders, # *p* < 0.01, vs. OIT-responders.

To determine whether high serum levels of ferritin are predictive of hyporesponsiveness to iron supplementation, serum levels of ferritin at 0 and 6 months after the initiation of iron supplementation were compared among the OIT-responders, IIT-responders, and IIT-nonresponders. Serum levels of ferritin at 6 months were significantly higher in the IIT-nonresponders (299.5 ± 247.8 pmol/L) than in the IIT-responders 142.0 ± 69.9 pmol/L, *p* < 0.05) and the OIT-responders (99.8 ± 54.8 pmol/L, *p* < 0.01, Figure 6). At the end of the study, serum iron levels were adequate and not statistically significant in the three groups; 13.3 ± 5.8 μmol/L in the OIT-responders, 9.8 ± 5.4 μmol/L in the IIT-responders, and 11.2 ± 3.3 μmol/L in the IIT-nonresponders. Despite no statistical difference in serum levels of hepcidin-25 between the groups at the start of the study, serum hepcidin-25 levels tended to be higher in the IIT-nonresponders (8.1 ± 10.5 nmol/L) than in the IIT-responders (4.1 ± 5.0 nmol/L). Similarly, at 6 months after iron therapy, serum levels of hepcidin-25 tended to be higher in the IIT-nonresponders (8.5 ± 9.0 nmol/L) than in the IIT-responders (2.0 ± 2.0 nmol/L). In addition, TSAT improved in the OIT-responders (30.3 ± 13.6%) and 20.1 ± 12.5% in the IT-responders, whereas it remained low (15.2 ± 0.9%) in the IIT-nonresponders at the end of the study.

**Figure 7:**
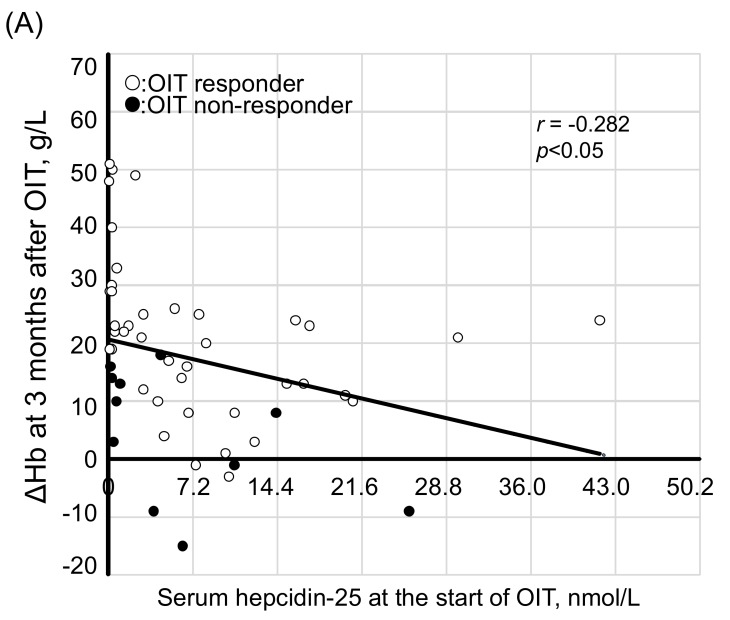

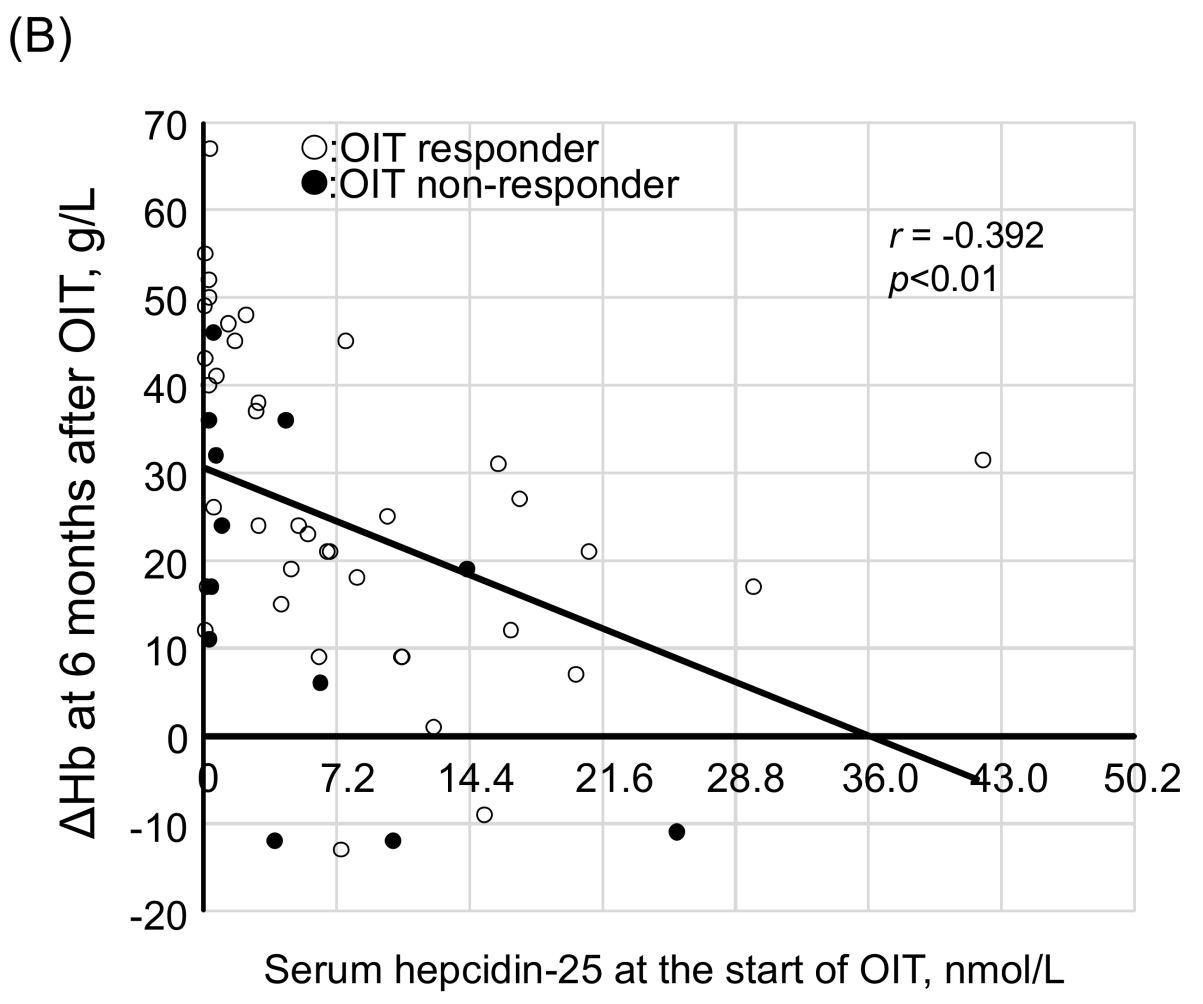
Correlation between serum levels of hepcidin-25 at the start of OIT and ΔHb at 3 and 6 months after OIT. Serum levels of hepcidin-25 at the start of OIT were negatively correlated with ΔHb at 3 months (*r* = −0.282, *p* < 0.05) (**A**) and at 6 months (*r* = −0.392, *p* < 0.01) (**B**) in 51 HD patients. The correlation equation was calculated by Pearson’s correlation coefficient test. White circle: OIT-responders, black circle: OIT-nonreponders.

We next examined whether the values for hepcidin-25 are predictive of the response to iron supplementation. In the 51 patients, serum levels of hepcidin-25 at the start of OIT were negatively correlated with ΔHb at 3 months (*r* = −0.282, *p* < 0.05, Figure 7A) and ΔHb at 6 months (*r* = −0.392, *p* < 0.01, Figure 7B), respectively. When correlation between the two parameters were determined only in the OIT-responders, a more significant correlation was noted between hepcidin-25 and ΔHb at 3 months (*r* = −0.525, *p* < 0.01) and 6 months (*r* = −0.578, *p* < 0.01).

**Figure 8:**
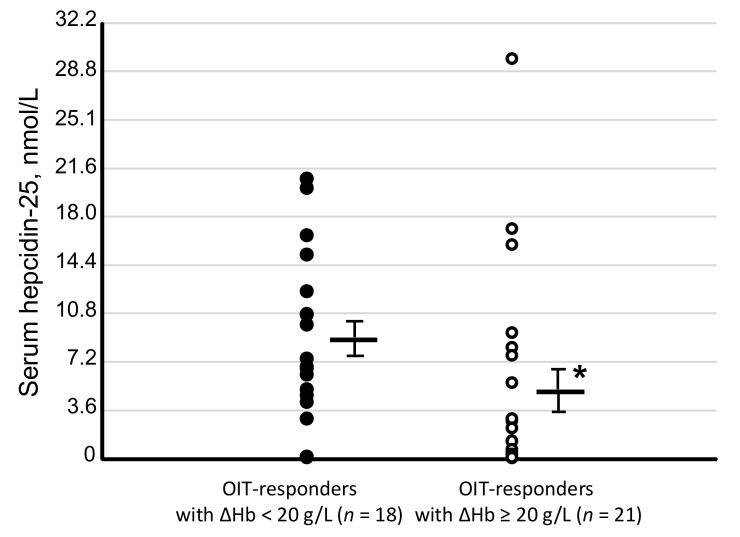
Serum levels of hepcidin-25 at the start of OIT between 21 OIT-responders with ΔHb ≥ 20 g/L and 18 OIT-responders achieving target Hb but ΔHb < 20 g/L at 3 months after OIT. Serum levels of hepcidin-25 at the start of OIT were significantly lower in the 21 OIT-responders with ΔHb ≥ 20 g/L than those in the 18 OIT responders who achieved target Hb (120–130 g/L) but ΔHb < 20 g/L. Comparison of two nonparametric data groups was analyzed by the Mann–Whitney U test. White circle: OIT-responders with ΔHb ≥ 20 g/L, black circle: the 18 OIT-responders with ΔHb < 20 g/L. * *p* < 0.05 vs. OIT-responders with ΔHb < 20 g/L.

Serum levels of hepcidin-25 were significantly lower in the 21 OIT-responders with ΔHb ≥ 20 g/L (5.1 ± 7.6 nmol/L, *p* < 0.05, Figure 8) than in the remaining 18 OIT-responders with ΔHb < 20 g/L (8.9 ± 2.2 nmol/L). Serum levels of hepcidin-25 at the start of OIT tended to be lower in the IIT-responders (*n* = 7, 4.1 ± 5.0 nmol/L) than in the IIT-nonresponders (*n* = 5, 8.1 ± 10.5 nmol/L). There was a similar trend for decreased serum levels of hepcidin-25 in the OIT-responders plus IIT-responders (*n* = 46, 4.6 ± 6.9 nmol/L) compared to those in the IIT-nonresponders (*n* = 5, 8.1 ± 10.5 nmol/L). 

Finally, we examined whether serum triglycerides can indicate the response to OIT due to higher serum triglycerides associated with the OIT-nonresponders (see Table 1). Despite a very weak negative correlation between serum triglycerides and hepcidin-25 (*r* = −0.319, *p* = 0.02), there was no significant correlation between serum triglycerides and ferritin (*r* = 0.224, *p* = 0.08) and ΔHb at 3 months (*r* = −0.08, *p* = 0.59).

There were no serious adverse effects associated with OIT or IIT and iron supplementation was well tolerated.

## 4. Discussion

There is a concern about a link between serum levels of ferritin and a risk of mortality in HD patients with IDA. Some investigators in the U.S. proposed that serum ferritin levels of 1123.5–2696.4 pmol/L were not associated with increased risk of mortality in HD patients receiving IIT and ESA if malnutrition and inflammation were controlled [27], while the same research group reported a trend for higher mortality in non-dialysis CKD patients with serum ferritin >561.8 pmol/L [28]. International guidelines for the management of IDA recommend that IV iron should be discontinued when serum ferritin is >1123.5–2696.4 pmol/L [3,29]. However, high levels of CRP [6,30] and serum ferritin of >179.8–1797.6 pmol/L were associated with worse outcome in HD patients [14,31,32,33,34]. In addition, serum ferritin levels of >1123.5–1797.6 pmol/L were associated with high mortality in HD patients in Europe, the U.S. [34,35] and Taiwan [31]. In these studies [31,34,35], the levels of CRP were high in the majority of the HD patients. In contrast, relatively lower serum ferritin levels (>179.8–1114.5 pmol/L) were associated with a significant risk of mortality in Japanese HD patients with minor inflammation receiving IIT and ESAs [14,32,33,36], suggesting that cutoff of serum ferritin to predict a risk of iron overload and mortality in HD patients may be lower in HD patients in the absence of inflammation. Aggressive IIT has been used in HD patients of Western countries probably due to high prevalence of inflammation, which increases serum ferritin and hepcidin-25, thereby inhibiting iron efflux and absorption for erythropoiesis and requiring higher dose of IV iron. In support of this finding, it was shown that if the CRP increased by 1 mg/L, possibilities to achieve tHb were reduced by 7.5% in HD patients with IDA [37]. High dose of IIT may increase a risk of infection-related mortality [38], cardiovascular events and high mortality in HD patients [12], although it was challenged [11]. On the other hand, low ferritin levels (<47.2–67.4 pmol/L) were also associated with a higher risk of mortality in HD patients receiving IIT and ESA [14,33], suggesting that both low and high serum ferritin are at a risk of mortality in HD patients.

The present study showed optimal serum ferritin levels of 67.4–89.9 pmol/L for the management of IDA with OIT in HD patients with minor inflammation. The serum ferritin levels in our patients are likely to represent more accurately iron status because of no overt inflammation. Further increment of serum ferritin was accompanied by increased levels of hepcidin-25 (see Figure 4), which can inhibit iron absorption and efflux, resulting in reduced iron availability for erythropoiesis and subsequent decrease in Hb levels [39]. This may explain a linear correlation between Hb and serum ferritin during 1–5 months in the OIT-responders, while no correlation was found when serum ferritin rose more than the threshold levels thereafter. In support of this finding, elevated iron indices failed to increase Hb in non-dialysis CKD patients with iron repletion (Hb > 110 g/L) [40]. The HD patients with serum ferritin of 67.4–179.8 pmol/L receiving IIT and ESA had better outcome than those with serum ferritin <67.4 pmol/L or >179.8 pmol/L [14]. The MRI study reported that optimal levels of serum ferritin were 359.5 pmol/L for liver iron content (LIC) >50 μmol/g (mild iron overload) and 651.6 pmol/L for LIC >200 μmol/g (severe iron overload) [10]. Thus, our optimal levels of serum ferritin in HD patients with minor inflammation receiving OIT and CERA are far less than these serum ferritin levels that might cause iron overload [10]. Our data support that therapeutic strategy for IDA in HD patients should include minimization of a risk of inflammation including infection that increases the required iron dose for IDA in HD patients [41].

Our study suggested that OIT was as effective as IIT in HD patients [23,24,25], that the response to OIT could reduce the dose of CERA in HD patients [8,24], and that in the absence of inflammation, low serum levels of ferritin and hepcidin-25 could be predictive of the response to OIT or IIT as reported in non-dialysis CKD [42] and our previous HD patients [25]. Iron absorption was not impaired in HD patients [43] but reduced when high inflammation was present [44]. This supports the efficacy of OIT for the management of IDA in our HD patients with minor inflammation. Of note is that the dose of OIT in our study is very low as compared to that in other studies showing similar benefit of OIT in HD patients [23,24]. Thus, in the absence of inflammation, low dose OIT may be adequate for the management of IDA in the majority of HD patients.

It remains elusive why some HD patients respond to OIT and others not. Inflammation is associated with obesity, diabetes mellitus and malnutrition, which are frequently seen in CKD patients. It is possible that these conditions could affect the response to iron therapy by increasing ferritin and hepcidin-25. In fact, increased levels of ferritin and hepcidin-25 were associated with obesity [45] and malnutrition [46]. In our study, however, no difference was found in prevalence of these conditions between the OIT-responders and the OIT-nonresponders. Our study confirmed our previous finding that ferritin and hepcidin-25 could be predictive factors for the response to OIT in HD patients in the absence of inflammation [25]. Serum levels of hepcidin-25 were positively correlated with triglycerides and interleukin (IL-6) and CRP in HD patients [47,48]. In addition, high serum levels of triglycerides were associated with hyporesponsiveness to IIT in HD patients [19]. Despite high levels of serum triglycerides in the OIT-responders compared to the OIT-nonresponders, no correlation was found between serum triglycerides and ferritin and ΔHb in our patients. Thus, it remains to be determined whether serum triglycerides is a predicting factor of the response to iron therapy in HD patients. Our study also showed that the IIT-nonresponders were associated with increased serum ferritin and adequate serum iron, whereas TSAT remained low at the end of the study, suggesting that the IIT-nonresponders may have non-inflammatory FIDA [49]. If this is the case, a more dose of IV iron may be required for the management of FIDA in these patients. 

As other causes that may affect the response to iron therapy, reactive oxygen species (ROS) generation, reduced anti-oxidants, and increased IL-6 were associated with the HD patients who had even normal CRP [50,51]. These factors can increase ferritin and hepcidin-25, leading to reduced iron availability for erythropoiesis [39]. In fact, serum levels of IL-6 were correlated with those of ferritin and hepcidin-25 in CKD patients [17,46,48]. Regardless of ferritin and inflammation markers (CRP and IL-6), the levels of anti-oxidant glutathione peroxidase in erythrocytes were lower in the IIT-nonresponders than in the IIT-responders [52]. In HD patients, serum and erythrocyte folate concentrations were inversely correlated with serum ferritin in the IIT-responders [53]. CKD is associated with hypoxia which can increase hypoxia-inducible factor (HIF). HIF prolyl hydroxylase (HIF-PHD) inhibitors, which stabilize HIF, can increase Hb by inhibiting hepcidin-25 regardless of iron status in HD patients [54]. In fact, urine HIF-α mRNA was increased in CKD patients than controls [55]. Further studies are needed to determine the predictive values of these factors for the response to iron therapy in HD patients.

Little is known about early change of serum ferritin following iron supplementation in CKD patients. Our study demonstrated that serum ferritin first fell and started to rise at 2–3 months following OIT in HD patients. Kapoian et al. showed that despite an early increase in Hb levels, a decrease in ferritin levels was noted in HD patients at 1 month following IIT [56]. In CKD patients treated with oral ferric citrate or liposomal iron, decreased or unchanged serum ferritin was noted at 1 month after iron therapy [57]. In support of our finding, serum ferritin started to rise at 3 months after oral ferric citrate in HD patients and a decrease in the rate of rise of ferritin was noted among subjects on ferric citrate, probably due to stability of intestinal absorption of iron [58]. 

Although serum ferritin quickly rose after IIT in CKD patients due to direct infusion of iron into vessels [57], intestinal iron absorption after OIT was impaired in HD patients as compared to healthy controls [44]. Intestinal iron absorption became stable at 4 months after OIT in HD patients [59]. Bone marrow response to iron is limited to 20 mg/day of elemental iron, and an increase in Hb of 1 g/dL occurs every two to three weeks on iron therapy [60]. However, it may take up to 4 months for the iron stores to return to normal after the Hb has corrected [60]. In addition, intestinal absorption of oral iron fumarate used in our study is lower than oral ferrous sulphate [61]. In support of these findings, in healthy individuals receiving OIT after blood donation, serum ferritin recovered after 107 days [62]. These observations may explain a transient decrease in serum ferritin at early phase after starting OIT despite an increment of Hb in HD patients.

Finally, our study suggested that low dose of IIT could be a second line of iron supplementation for IDA in HD patients with minor inflammation who were resistant to OIT. IIT with ferrous saccharated (300–800 mg bolus once a month followed by 50 mg weekly for 3 months) were beneficial for IDA in 13 (76.4%) of the 17 HD patients who failed to maintain the tHb (10–11 g/dL) after the treatment with OIT and ESA [63]. However, higher dose of IV iron could increase a risk of systemic inflammation, cardiovascular events, infection and mortality in HD patients through iron overload-induced immune dysfunction, generation of reactive oxygen species and mitochondrial dysfunction [1,11,22]. Total doses of IIT in our protocol is lower than the low-dose maintenance IIT (31.25 mg/week over 1 year) that failed to prevent a risk of iron overload in HD patients with moderate anemia [64]. Our protocol (OIT and IIT) were well tolerated probably due to the low dose of iron used. However, the IIT-nonresponders who are likely to have non-inflammatory FIDA [49] may require higher dose of IV iron for the management of anemia.

## 5. Conclusions

OIT is beneficial for the management of IDA in Japanese HD patients with minor inflammation. Optimal levels of serum ferritin appear to be 67.4–89.9 pmol/L when tHb is 120–130 g/L, and further increment of serum ferritin is accompanied by increased levels of hepcidin-25, which inhibits iron availability for erythropoiesis, resulting in subsequent decrease in Hb. IIT can be a second choice of treatment for IDA in HD patients who are resistant to OIT. Limitations of our study include a small sample size and exclusion of HD patients with high inflammation and those with FIDA. Therapeutic strategy for IDA should be different among HD patients with high inflammation and those without, and include minimization of a risk of inflammation that increases ferritin and hepcidin-25, leading to hyporesponsiveness to iron therapy. Further studies using a large number of HD patients would be necessary to determine the benefit of OIT, optimal levels of serum ferritin to avoid a risk of iron overload, the benefit of IIT in patients who are resistant to OIT, and whether the response to iron therapy is different in HD patients with and without inflammation as well as whether predictive values of ferritin and hepcidin-25 for the response to iron therapy are dependent on inflammation.

## Figures and Tables

**Table 1:** Demographic and laboratory data in OIT-responders and OIT-nonresponders.

	OIT-Responders (*n* = 39)	OIT-Nonresponders (*n* = 12)	*p* Value
Age (years)	66.0 (18.0)	62.5 (13.8)	0.46
Female (%)	52	33	0.32
Body mass index (kg/m^2^)	21.3 (4.0)	21.9 (3.6)	0.84
HD vintage (years)	4.5 (10.5)	4.0 (7.8)	0.50
spKt/V	1.49 (0.4)	1.35 (0.59)	0.65
Hb (g/L)	10.3 (1.4)	9.2 (1.3) *	0.04
MCV (fL)	84.8 (8.1)	85.9 (8.6)	0.49
Serum ferritin (pmol/L)	39.8 (51.2)	29.2 (53.4)	0.47
Serum iron (μmol/l)	12.5 (10.7)	8.3 (8.2)	0.13
TSAT (%)	18.2 (14.5)	15.5 (13.8)	0.71
Serum hepcidin (nmol/L)	5.1 (10.1)	2.8 (6.7)	0.25
Serum creatinine (μmol/L)	1034 (309)	919 (265)	0.50
Serum albumin (g/L)	35 (5)	36 (2)	0.34
Serum triglycerides (mmol/L)	0.95 (0.44)	1.42 (0.96) *	0.02
Serum calcium (mmol/L)	2.4 (0.1)	2.4 (0.4)	0.35
Serum phosphorus (mmol/L)	1.7 (0.6)	1.8 (0.5)	0.66
i-PTH (ng/L)	62.0 (82.0)	50.5 (84.5)	0.62
CRP (mg/L)	0.4 (0.9)	0.6 (0.8)	0.69
CERA dose (μg/week)	150 (50)	150 (62.5)	0.73
Comorbidities (%)			
Diabetes mellitus	32	33	
Hypertension	85	67	
Coronary artery disease	26	25	
Congestive heart failure	0	0	
Vascular disease	8	8	

Data are expressed as median (interquartile). OIT, oral iron therapy; CRP, C-reactive protein; HD, hemodialysis; CERA, continuous erythropoietin receptor activator; i-PTH, intact parathyroid hormone; MCV, mean corpuscular volume; TSAT, transferrin saturation. Comparison of two nonparametric data groups were determined by the Mann-Whitney U test. * *p* < 0.05, vs. OIT responders.
